# Cultivating antimicrobial resistance: how intensive agriculture ploughs the way for antibiotic resistance

**DOI:** 10.1099/mic.0.001384

**Published:** 2023-08-22

**Authors:** Matthew Kelbrick, Elze Hesse, Siobhán O' Brien

**Affiliations:** ^1^​ Department of Evolution, Ecology and Behaviour, Institute of Infection, Veterinary and Ecological Sciences, University of Liverpool, Crown Street, Liverpool, L69 7ZB, UK; ^2^​ College of Life and Environmental Science, University of Exeter, Penryn, Cornwall, TR10 9FE, UK; ^3^​ Department of Microbiology, Moyne Institute for Preventive Medicine, School of Genetics and Microbiology, Trinity College Dublin, Dublin 2, Republic of Ireland

**Keywords:** Anthropocene, AMR, antibiotics, evolution, cross-resistance, agriculture, soil

## Abstract

Antimicrobial resistance (AMR) is a growing threat to public health, global food security and animal welfare. Despite efforts in antibiotic stewardship, AMR continues to rise worldwide. Anthropogenic activities, particularly intensive agriculture, play an integral role in the dissemination of AMR genes within natural microbial communities – which current antibiotic stewardship typically overlooks. In this review, we examine the impact of anthropogenically induced temperature fluctuations, increased soil salinity, soil fertility loss, and contaminants such as metals and pesticides on the *de novo* evolution and dissemination of AMR in the environment. These stressors can select for AMR – even in the absence of antibiotics – via mechanisms such as cross-resistance, co-resistance and co-regulation. Moreover, anthropogenic stressors can prime bacterial physiology against stress, potentially widening the window of opportunity for the *de novo* evolution of AMR. However, research to date is typically limited to the study of single isolated bacterial species – we lack data on how intensive agricultural practices drive AMR over evolutionary timescales in more complex microbial communities. Furthermore, a multidisciplinary approach to fighting AMR is urgently needed, as it is clear that the drivers of AMR extend far beyond the clinical environment.

## Introduction

**Fig. 1.**
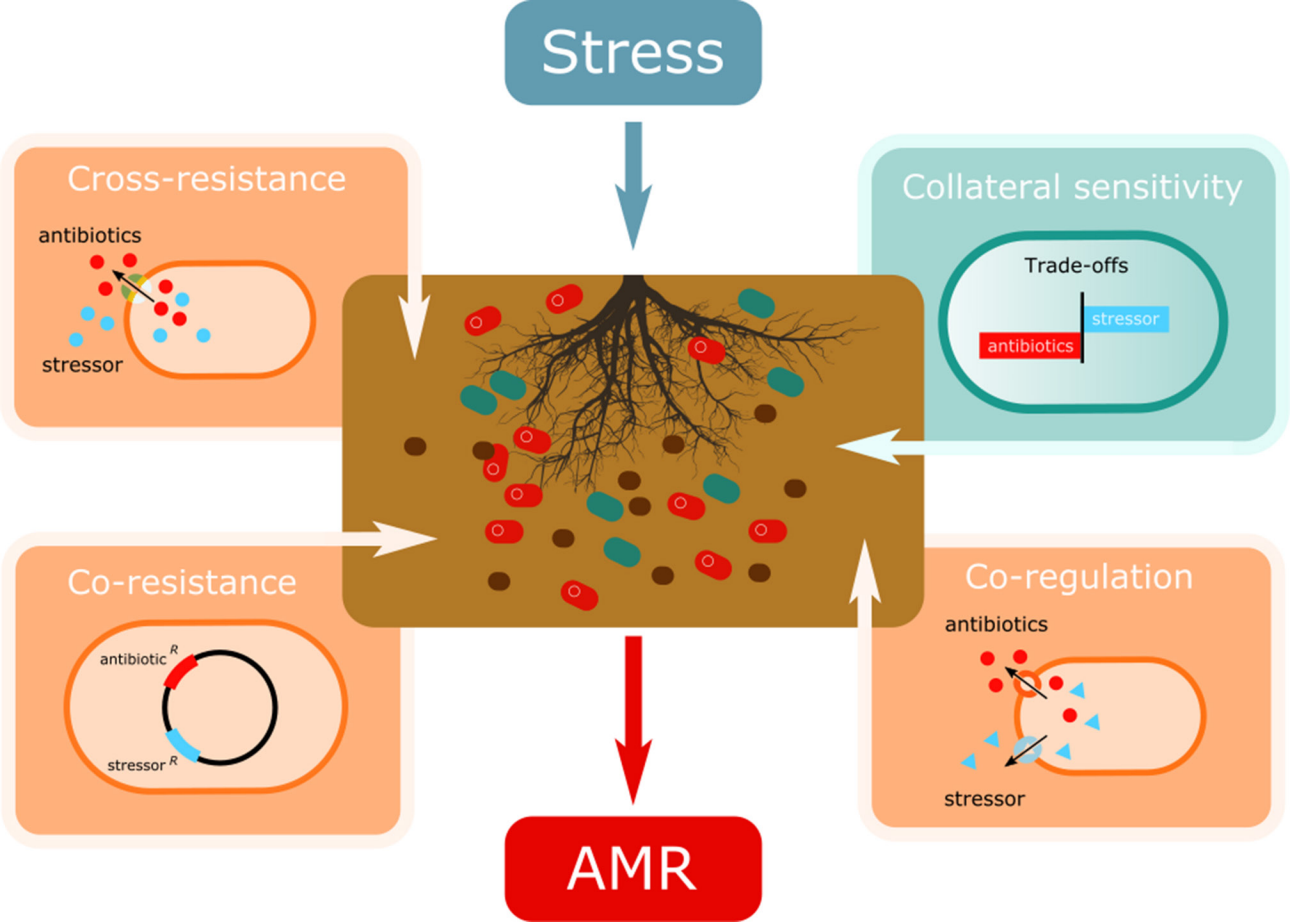
Illustration of the eco-evolutionary mechanisms at play in intensively farmed soils which can shape the dissemination of antimicrobial resistance (AMR) in microbial communities. Cross-resistance and co-regulation occurs when stress-resistance mechanisms inadvertently upregulate AMR genes either directly (cross-resistance) or indirectly (co-regulation). The spread of mobile genetic elements (MGEs) in soil bacterial communities exposed to stress can also cause AMR dissemination since stress-resistance and AMR genes are often present on MGEs (co-resistance) [15]. Finally, trade-offs between resistance mechanisms could instead constrain AMR evolution when selection for resistance against one stressor increases sensitivity to a second stressor [91].

Antimicrobial resistance (AMR) is a pressing global issue expected to cause more than 10 million deaths annually in 2050 [1, 2]. While responsible antibiotic stewardship has been advocated as crucial for controlling AMR, emerging evidence suggests that reducing antibiotic use alone may not be sufficient for curbing [3–5] or reversing AMR [6]. The prevailing paradigm suggesting that antibiotic resistance is metabolically costly is being increasingly challenged because compensatory mutations and genetic co-selection can negate the cost of resistance in natural bacterial populations [6]. Moreover, environmental stressors such as pesticides [7], increasing temperatures [8] and heavy metal contamination [9] could make AMR genes beneficial – even in the absence of antibiotics. Worryingly, this suggests that increasingly intensive agricultural practices (Fig. 1) could drive selection for AMR, potentially compromising the efforts of antibiotic stewardship programmes [10]. In this review, we discuss how anthropogenic activities, particularly intensive agricultural practices, could be important yet overlooked drivers of AMR.

**Fig. 2.**
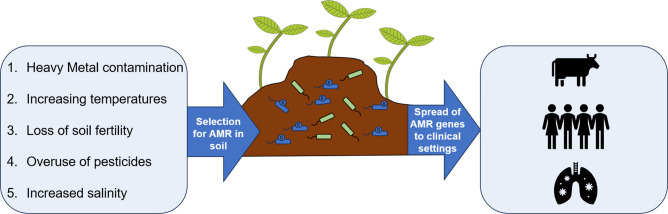
Anthropogenic stressors such as heavy metal contamination, pesticide application, increased salinity, loss of soil fertility and increasing temperatures could be key drivers of AMR dissemination in intensively farmed agricultural sites. Intensive agriculture could act as a reservoir of AMR genes, which can then subsequently spread to animals and humans.

Intensive agricultural practices have been highlighted as significant drivers of AMR [11], with conventionally farmed sites harbouring more AMR genes than organically farmed sites [12]. Intensively farmed soils and their constituent microbial communities are frequently exposed to anthropogenically induced stressors, such as agricultural chemicals and pollutants [11]. Such stressors can drive AMR in three key ways (Fig. 1). First, stressors can select for stress-resistance mechanisms in bacteria that cause cross-resistance to antibiotics (Fig. 1). For instance, efflux pumps are often upregulated in response to heavy metals but can also expel a wide range of clinical antibiotics [13]. Second, environmental stress can select for mutations in global stress regulators, which can have myriad effects on the cell, including enhanced AMR [14]. Third, stressors can accelerate the spread of AMR mobile genetic elements (MGEs), when stress and antibiotic resistance genes are located on the same MGE (co-resistance; Fig. 1) [15]. Together, this suggests that agricultural fields may serve as reservoirs of AMR which could spread through the food chain and into clinical settings (Fig. 2). In this review, we highlight five key ways in which intensive agricultural practices amid climate change could drive AMR. We discuss how, even without antibiotic exposure, intensive agricultural practices could independently drive the emergence of AMR through physiological responses, *de novo* evolution, species sorting toward resistant taxa and enhanced horizontal transfer of AMR genes.

## Metal contamination

Mining is a major cause of land degradation, having left a substantial historical and contemporary global footprint [16]. For example, it has been estimated that an area of ~1 million km^2^ globally is covered by mine waste, a large proportion of which is located in populated areas [17]. Other important sources of environmental metal pollution include agriculture and atmospheric deposition resulting from industrial processes [18]. In particular, practices associated with intensive agriculture [19], such as the application of biocides (e.g. copper), manure and sewage sludge [20], are often a local source of environmental metal pollution.

While many metals are essential to biological functions, most are toxic at high concentrations [20]. Hence, the release and remobilization of non-degradable metals into the environment can pose a serious long-term threat to ecosystem- and human-health [21]. For example, consumption of crops and other food products harvested from metal-polluted environments can negatively impact human health via trophic cascades in the food chain (e.g. arsenic accumulation in paddy rice [22]; mercury accumulation in freshwater fish [23]). Metal contamination can also indirectly impact human health by co-selecting microorganisms that are resistant to antibiotics (AMR) [24], even in the absence of antibiotics themselves [25]. For example, using pot experiments, a recent study found enhanced levels of antibiotic resistance genes (ARGs) in soil communities fertilized with swine slurry compared to non-treated soils. Importantly, metals were a key factor in driving the observed increase in ARGs in the resident soil communities [26]. A recent metagenomic study confirms these findings: the addition of zinc oxide (a growth promotor in animal husbandry) to pig feed led to increased co-occurrence of metal and antibiotic resistance genes in pig faeces and caused the dissemination of these genes into the wider agricultural environment [27]. Similarly, in terrestrial subsurface soils, heavy metal content was found to be a better predictor of ARG content than bacterial community composition, MGEs or physico-chemical factors [28]. Because of its clinical and environmental importance, there is a large body of work on the co-selection of metal and antibiotic resistance [9, 19, 29, 30]. These studies have revealed that different mechanisms can underpin the co-selection process, having important implications for the acquisition and spread of AMR in natural environments.

Bacteria have evolved a wide range of mechanisms to cope with toxic metals [31–35]. Some of these mechanisms confer cross-resistance to other toxic compounds. Examples of cross-resistance mechanisms include multi-drug resistant (MDR) efflux pumps [13] and several factors reducing cell permeability and the influx of compounds (e.g. biofilm formation [31]; downregulation or deletion of outer-membrane porins [36, 37]). For instance, bacteria commonly encode (sometimes multiple) MDR efflux pumps that can expel a wide range of compounds, including clinical antibiotics. However, efflux of clinical antibiotics is often a side-effect of the primary role of MDR efflux pumps [24] – protection against natural toxins (e.g. metals, bile salts, aromatic compounds and quorum-sensing molecules) and host colonization [38]. The presence of xenobiotics in agricultural soils (e.g. metals, pesticides) can therefore selectively favour bacteria that harbour, or overexpress, MDR efflux pumps, thereby inadvertently selecting for increased resistance to clinical antibiotics. Indeed, the expression of cross-resistance mechanisms, such as efflux systems and biofilms, often changes in response to abiotic stress [9, 24, 39].

Bacteria can also harbour resistance mechanisms that specifically target toxic metals, including metal reduction and extracellular sequestration. For example, most bacteria produce metal-chelating siderophores [40, 41]. Bacteria release these compounds into the environment, where they bind to toxic metals, preventing them from being taken up and killing bacterial cells [42, 43]. The production of siderophores is typically upregulated in response to toxic metal stress, both at the species level [44] and community level (i.e. ecological species sorting favouring siderophore-producing taxa [45, 46]). Crucially, while siderophores do not confer increased antibiotic resistance, their production can increase pathogen virulence. The fact that siderophores are potent iron chelators enables bacteria to scavenge poorly soluble iron from their host and be taken up by cells carrying specific outer-membrane receptors [47]. A recent study confirms this notion showing that copper-mediated selection favours higher levels of siderophore production in the opportunistic pathogen *Pseudomonas aeruginosa *, thereby increasing pathogen virulence [48].

Some metal resistance genes do not provide cross-resistance to other toxic compounds but are associated with AMR because they are co-located on the same chromosome or MGE (i.e. co-resistance [9]). Importantly, resistance genes are often enriched on MGEs [33], including plasmids that spread horizontally by conjugation. Such horizontal gene transfer (HGT) is considered to be a major evolutionary force [49, 50] and is thought to play a vital role in the spread of AMR genes, both within and across bacterial pathogen species [24, 51, 52]. Previous work has shown that the presence of metals in the environment can enhance the horizontal spread of plasmids [53–55], including those that harbour both AMR and metal-resistance genes. For example, genes encoding aerobactin – a metal-chelating siderophore – and AMR are located on the same plasmid in *Escherichia coli* strains isolated from sewage [56]. Co-location could then subsequently enhance the dissemination of AMR genes in environments where the production of siderophores is selectively favoured (i.e. in iron-deficient or metal-polluted environments).

Heavy metals continue to be used routinely for both crop and livestock production, although the use of some heavy metals as antimicrobials (e.g. zinc oxide) was banned in the EU in June 2022 [Regulation (EU) 2019/6 on Veterinary Medicinal Products]. A recent scoping review article by Anedda *et al*. [57] examined 73 studies addressing the impact of heavy metal contamination on ARG dissemination [57]. Despite differences in objectives, sample types, locations and methods between these studies, they all asserted a clear link between heavy metals and AMR in the primary food production environment. Together, this is strong evidence to support better management of heavy metal use in agriculture, including legislation that supresses the use of heavy metals or heavy-metal-containing fertilizers in routine farm management.

## Increasing temperatures

Anthropogenic activity is warming the climate at an unprecedented rate, with an average projected increase of 1.5–2 °C in the coming decades [58]. Extreme temperature events, warmer nights and more variable precipitation will significantly reduce agricultural crop yield and expand the potential habitable range of some insect and disease pests [59]. Increasing global soil temperatures and associated drought can similarly shape soil microbial community composition, diversity and functioning, disrupting microbe–plant feedback [60–62]. However, far less is known about how increasing temperatures can alter the evolutionary trajectory of soil microbial populations per se, and the impact such evolved changes can have on microbe–plant interactions. There is solid evidence to suggest that the global burden of AMR is positively correlated with increased local temperature, suggesting that even minor temperature increases could influence the evolution and spread of AMR in soil microbial communities [63].

Since the origin of life, microbes have been faced with temperature stress. In fact, microbial pathogens may have been the driving force behind the evolution of warm-blooded animals, as fever would be more effective at repelling infections in warm-blooded versus cold-blooded animals [64]. Temperatures exceeding the optimum temperature for bacteria (*T*_opt_) can cause cellular proteins to misfold, damage DNA and RNA, and increase membrane fluidity [65]. Bacteria can cope with short-term temperature extremes via transient heat shock responses (HSRs) [65, 66]. In *E. coli,* the HSR is regulated by the alternative sigma factor σ32 [67], characterized by reduced growth rates and upregulation of heat shock proteins, such as (i) chaperones to prevent protein misfolding (e.g. ClpB, DnaK, DnaI and GroEL/ES); (ii) proteases to degrade misfolded proteins (e.g. ClpP and ClpX); (iii) DNA/RNA repair enzymes; (iv) metabolic enzymes; (v) outer membrane stability proteins; and (vi) membrane transport proteins [68]. This HSR is highly conserved and allows cells to temporarily counteract the effects of short-term stress by slowing down growth and re-directing resources into preventing DNA damage.

It has been recently suggested that mechanisms of AMR are co-opted from such stress responses to temperature [8, 69]. Different classes of antibiotics can simulate heat stress, or cold stress, depending on the class of antibiotic. For example, using 2D gel electrophoresis, it has been shown that treating *E. coli* with aminoglycosides (antibiotics that target the 30S ribosomal subunit) results in protein expression changes that are ‘virtually indistinguishable from that produced by a shift in temperature*’* from 28 °C to 42 °C [70]. More recently, Cruz-Loya *et al.* [8] used stressor interaction networks to reveal that *E. coli* physiological responses to low or high temperatures are clearly separated, and each is grouped with particular antibiotics that have similar effects to cold or heat respectively. For example, aminoglycosides, as well as nitrofurantoin and trimethoprim, all have similar physiological effects to heat stress (44–46 °C), while macrolides, tetracycline and fluoroquinolones emulate cold stress (22–37 °C). While the exact causes of this interaction similarity are unclear [71] it was hypothesized that translational misreading caused by both heat and aminoglycoside antibiotics warrant a similar protective response by the cell. For example, heat shock chaperones (DnaK and GroEL) protect cells against aminoglycoside antibiotics by preventing protein misfolding and aggregation in *Acinetobacter baumanii* and *E. coli* [49]. Moreover, the deletion of *cspB* – associated with the cold-shock response – can lead to an enhanced HSR and consequently increased resistance to heat-similar aminoglycosides and trimethoprim [50]. Together, this suggests that as temperatures increase under climate change, particular classes of antibiotics may be more vulnerable to resistance evolution (i.e. heat-similar antibiotics, such as aminoglycosides). Conversely, resistance toward cold-similar antibiotics (e.g. tetracycline and chloramphenicol) could become more costly [72].

Despite the potential for temperature stress to influence AMR via activation of the HSR, it is less clear whether temperature-similarity profiles feed into selection over evolutionary time. The HSR is generally transient (in the order of magnitude of minutes) in *E. coli *, and hence is fitting for short-term rather than long-term temperature extremes. However, activation of the HSR could ‘prime’ bacterial populations for resistance before a heat-similar antibiotic is applied [73]. In this case, cells with an active HSR would gain a slight advantage over non-expressing cells since they would be better equipped to deal with misfolded DNA. Even such a short-term physiological advantage could expand the window of opportunity for resistance mutations to evolve.

A tour de force by Rodríguez-Verdugo and colleagues [8, 71, 74] provides unique insights into how bacteria might adapt to heat stress over evolutionary time and how this could impact AMR. Rodríguez-Verdugo *et al.* [74] compared the mid-exponential phase gene-expression profile of *E. coli* growing at 37 and 42 °C, revealing differential expression of 1737 genes between the two temperature treatments. Downregulated genes at 42 °C included ribosomal constituents (*rpl*, *rpm* and *rps*) involved in translation, amino acid biosynthesis, flagellum motility and ribonucleoside biosynthesis. Perhaps most surprisingly, however, is that growth at 42 °C resulted in the downregulation of heat shock proteins involved in the HSR, including subunits of core RNA polymerase (RNAP) *rpoA*, *rpoB* and *rpoC*. Similarly, most HSR encoding chaperones were also downregulated (*clpB*, *dnaJ*, *groEL* and *groES*) at 42 °C. This result was explained by the fact that the gene-expression profiles were assayed at the mid-exponential phase, at which point the HSR was already turned off in this particular experimental setup. This suggests that the transient HSR might play a minimal role in priming populations for AMR. However, further experiments are needed to test if a short-term advantage could enhance selection for resistance over longer timescales.

Rodríguez and colleagues also experimentally evolved 114 *E. coli* populations under thermal stress (42.2 °C for 2000 generations) [74]. While initially, the general stress response system was activated (slowing down growth and reducing gene expression of RNAP, increasing transcriptional efficiency at 42 °C), this heightened stress response was not maintained throughout the experiment. Conversely, after 2000 generations, mutations in the β-subunit of RNAP (*rpoB*) instead restored growth and allowed the cell to revert to a gene expression profile similar to the ancestral (pre-stressed) state [71]. In 12 populations, resistance was mediated by single SNPs in codon 572, causing amino acid substitutions in *rpoB* and incidentally conferring rifampicin resistance ranging from ×10 to ×320 higher than susceptible cells. Using constructed *rpoB* mutants with SNPs in codon 572 they confirmed that this particular *rpoB* mutation (i) conferred high levels of rifampicin resistance via alterations in RNAP, and (ii) increased growth relative to the wild-type at 42 °C. Their work shows that the interplay between the costs and benefits of bacterial stress responses across multiple timescales can dictate the spread of AMR, at least in *E. coli.*

While the HSR could allow bacteria to tolerate short-term temperature extremes, more moderate increases in temperature could drive AMR evolution in multiple ways. Higher temperatures can have mutagenic effects by increasing replication errors and causing DNA damage [75]. Increased mutation rates could enhance the standing genetic diversity of populations, which, on exposure to antibiotics, could accelerate selection for resistance. Indeed, the evolutionary speed hypothesis (ESH) demonstrates that higher temperatures lead to faster evolutionary processes by increasing mutation rates and accelerating natural selection [76]. Temperature could also modulate HGT of AMR genes. MacFadden *et al.* [63] found that in the USA, an increase in daily minimum temperature of 10 °C (which is conceivable for some parts of the world by the end of the century) correlated with increased levels of AMR *in E. coli, Klebsiella pneumoniae,* and *Staphylococcus aureus *. Although the mechanism was not elucidated, it was speculated that increased temperatures accelerate HGT of AMR genes. Similarly, Reverter *et al*. [77] analysed data from 40 countries to pinpoint the key predictors of ARG frequencies in aquaculture [77]. They found a negative relationship between a country's vulnerability index (CVI; lower scores=higher vulnerability) and ARG abundance. Notably, this association was underpinned by the physical component of the CVI score, which encompassed mean temperature, water availability and frequency of extreme weather events. Together, these findings emphasize the deadly combination of AMR and climate change, where increasing temperatures could worsen the already growing crisis of AMR.

## Loss of soil fertility

The nutrient content of natural soils is declining on a global scale [78]. Conversion of native vegetation and land to intensive agricultural fields results in substantial losses of C, N, P and S, which in turn reduce the nutrient content of food crops [78]. The causes of nutrient loss via conventional farming are multifaceted, including intensive and continuous crop cultivation, soil erosion, leaching, and removing or burning stubble [79]. Crop production is now heavily dependent on artificial and organic fertilizers to replenish the low nutrient content of soils.

While the impact of soil fertility loss on plants is now evident [79], we know far less about how nutrient loss might influence soil microbes directly. In soil, scarcity of resources as well as competition with other species can heavily limit bacterial growth. Soil bacteria spend most of their lives under nutrient limitation in long-term stationary phase [80], with short pulses of nutrient input (feast/famine dynamics) [81, 82]. However, as levels of C and N are depleted in intensively farmed soils, it is likely that bacteria will spend increasingly longer proportions of their lives in stationary phase, with selection to enhance long-term survival in an increasingly nutrient-limited environment. Worryingly, studies examining short-term microbial responses to starvation and long-term adaptation under nutrient limitation suggest that AMR and loss of soil fertility could be inextricably linked [83–85].

When nutrients are scarce, physiological responses by bacterial cells can permit long-term survival. Activation of the stationary phase sigma factor S (*σ* ^s^) stimulates key physiological changes, including (i) genome compaction, (ii) reduced membrane permeability, (iii) increased production of osmoprotectants and (iv) decreased expression of growth-promoting genes [85]. In *E. coli *, *rpoS* mutations conferring *σ* ^s^ overexpression can also have consequences for AMR – for example, a mutation in *rpoS* (87 D-6) can cause resistance to nalidixic acid [14]. Studies examining the starvation response of *E. coli* through prolonged incubation on agar plates also reveal the predictable emergence of rifampicin-resistant mutants, despite incubation taking place in the absence of antibiotics [14]. This finding was classically explained by stress-induced mutagenesis – where resistance emerges as a by-product of an increased mutation rate [86]. However, it is now acknowledged that rifampicin resistance is beneficial per se under starvation due to the pleiotropic effects of mutations in RNAP *β-*subunit (*rpoB*) that enhance fitness under starvation while incidentally conferring rifampicin resistance [83]. Mutations in *rpoB* are also the cause of high-level *β-*lactam resistance in methicillin-resistant *Staphylococcus aureus* [87]. Similarly, under nutrient-limitation*, Salmonella enterica rspL* mutants fail to induce the stationary phase sigma factor σ^s^, causing them to outcompete the slow-growing wild-type. These *rspL* mutations also enhance resistance to streptomycin [84]. Interestingly, these dynamics are reversed under nutrient-rich conditions, so the antibiotic-susceptible wild-type outcompetes *rspL* mutants. This result is presumably due to poorer translational fidelity of the mutant relative to the wild-type when nutrients are rich [84].

While stress response systems may drive AMR under nutrient limitation in the short term, experimental evolution approaches can reveal whether such responses will probably feed into long-term selection dynamics. One experimental evolution study of *E. coli* in laboratory media (in the absence of antibiotics) reported reduced susceptibility to erythromycin (2–4× MIC), fosfomycin (2–6× MIC), rifampicin (2–32× MIC) and streptomycin (2–3× MIC) after 500–1000 generations of evolution [83]. AMR genes evolved more frequently in low-nutrient versus high-nutrient media, reaching high frequencies and exhibiting extensive parallelism within the same treatment, indicating a strong selective advantage under nutrient limitation. Similarly, experimental evolution of *P. aeruginosa* in M9 minimal media found that under nutrient-limiting (but not nutrient-rich) conditions, *lasR* mutations evolved that caused enhanced levels of antibiotic resistance (possibly via increased expression of efflux pumps) [88, 89]. Interestingly, *P. aeruginosa lasR* mutants are common in clinical settings, and typically display enhanced resistance to tobramycin [90]. While such studies suggest that manipulating soil nutrient content could be a potential avenue for AMR control, we lack experiments that test how soil nutrients drive AMR in natural communities. In natural communities, resource competition can impose further constraints on the allocation of resources towards different traits/functions. This constraint can lead to trade-offs where adaptations evolved to optimize one trait are accompanied by a reduction in another [91]. Trade-offs can select against costly resistance mechanisms (e.g. porins keep out antibiotics but potentially also necessary nutrients) and constrain the evolution of multi-drug resistance [92]. However, the impact of soil fertility loss on AMR in natural microbial communities remains unexplored.

The loss of soil fertility is a complex problem, but solutions are relatively easy to implement. The EU Green Deal is encouraging the farming sector to adopt more sustainable methods, such as crop rotation, cover cropping, reduced tillage and better irrigation [93]. These approaches will have the dual effect of reducing emissions while restoring soil fertility. Still, further research is needed into the long-term benefits of such practices on the dissemination of ARGs in soil microbial communities.

## Overuse of pesticides

Intensive agricultural practices have led to cultivating densely packed crop monocultures to make the most economical use of space. Unfortunately, these conditions also facilitate the success of ‘pests’ such as fungi, bacteria, viruses, insects, arachnids and rodents, as well as undesirable vegetation (i.e. weeds) [94]. To mitigate crop death and prevent famine, it has become routine to apply pesticides. Pesticides are biocidal or biostatic compounds used to prevent and treat agricultural pest infestations. These pesticides are grouped into classes based on their target organism; for example, fungicides target fungi, and herbicides target undesirable vegetation.

Despite being designed to target specific pests (i.e. fungicides ‘only’ target fungi), many pesticides have been shown to have harmful effects on humans and the broader ecosystem [95]. Policies are now being implemented to rectify this emerging issue; for example, the European Union has recently banned some neonicotinoid pesticides due to their harmful effects on bees [96]. However, the impact of pesticides is not constrained to larger organisms; some fungicides have been recently found to inhibit the growth of soil bacteria [97, 98]. For example, pesticides, including the fungicides azoxystrobin and flutriafol, have been linked with the decreased abundance of nitrifiers which can reduce soil fertility and health [99]. Furthermore, the herbicide bromoxynil has been shown to inhibit the growth of soil bacterial populations, in turn reducing bromoxynil biodegradation and increasing fungicide persistence in soils [100]. It is now clear that pesticides can drive loss of soil microbial diversity, alter microbial soil community composition and even drive the rapid evolution of AMR in soil bacteria [7, 99, 100]. Despite the acknowledged non-target effects of pesticides and their potentially harmful implications on public health, they are still widely overused, further contributing towards the emerging AMR crisis.

The first line of defence by bacteria against pesticides is the downregulation of membrane porins or upregulation of efflux pumps to remove the chemicals from the cell [101, 102]. For example, the herbicide Dicamba has been shown to induce *soxRS* in *E. coli* [103]. The *soxRS* system can upregulate AcrAB efflux pumps which have been attributed to both pesticide and antibiotic resistances, including fluoroquinolones [104]. Copper-based fungicides can similarly drive the *de novo* evolution of mutations in the AcrAB-TolC multi-drug export pump, causing cross-resistance to tetracycline and chloramphenicol [105]. Mutations that cause overexpression of efflux pumps are a common early adaptation in response to both antibiotics and pesticides [15, 104, 106, 107] (cross-resistance; Fig. 1). This evidence suggests that pesticide-treated soils could act as a reservoir for AMR genes, which can ultimately return to the food chain (Fig. 2) [102].

Many bacteria can also degrade pesticides into less harmful residuals [108]. This biodegradation process can protect both the bacterium and other organisms from the stress of the pesticide. Therefore, such microbes and their enzymes have become areas of interest for bioremediation. One such group of enzymes are hydrolases (e.g. esterases, organophosphorus hydrolase and lipase) which can degrade certain chemicals through reactions with water. For example, organophosphorus insecticides are potent acetylcholinesterase inhibitors that are harmful to human health and other organisms [109, 110]. Bacterial-derived organophosphorus hydrolases are an effective means of bioremediation of these pesticides, as they reduce toxicity via the hydrolysis of phosphodiester bonds [109, 111–113]. The organophosphate degradation genes (*opd*) have been identified in a plethora of soil-dwelling bacteria such as *Flavobacterium *, *Pseudomonas *, *Bacillus* and *Agrobacterium* [114–116]. However, enzymatic modelling studies by Rangasamy *et al.* [117–119] have suggested that organophosphorus hydrolase from *Geobacillus* could dock with and hydrolyse streptomycin, ampicillin, chloramphenicol and cefotaxime. Furthermore, a plasmid-bound *α–β* hydrolase known to degrade organophosphate-degrading *α–β* hydrolases has been shown to reside on plasmids and provide resistance to a range of antibiotics [119].

Another group of enzymes involved in detoxification are glutathione *S*-transferases (GSTs). These isozymes are widely used by plants and insects as pesticide resistance mechanisms [120, 121]. GTSs have also been widely found in prokaryotes, and are associated with pesticide degradation in the rhizosphere [122, 123]. However, GTSs have also been associated with the degradation of antibiotics such as tetracycline, sulfathiazole and ampicillin [124]. Interestingly, some GTSs have also been shown to have peroxidase activity – an important family of enzymes which can degrade phenylamide herbicides [125]. Peroxidases and other pesticide-resistance mechanisms such as soxR can protect against oxidative (redox) stress. Since many antibiotics partly inhibit bacteria via the formation of reactive oxygen species (ROS) [126], it is likely that such pesticide-resistance mechanisms will similarly protect against antibiotics.

Finally, the co-occurrence of resistance genes to pesticides and antibiotics on MGEs poses a significant risk to public health, enabling the rapid dissemination of antibiotic resistance in bacterial populations. Studies have shown that exposure to certain pesticides can facilitate the transmission of AMR genes on MGEs in natural populations (i.e. co-resistance; Fig. 1). For instance, Liao *et al.* demonstrated that soils treated with glyphosate herbicides show an increased abundance of ARGs and MGEs compared to the non-herbicide-treated control [15]. These ARGs included genes associated with aminoglycoside, vancomycin, chloramphenicol and tetracycline resistance [15]. Similarly, azoxystrobin and carbendazim fungicides increase the expression of conjugation-related genes on plasmids, thereby increasing the spread of MGEs containing ARGs [127]. Pyrethroid-insecticides such as permethrin can also increase conjugation and mutation rates in *E. coli* [128]. Furthermore, a study of *E. coli* grown in a lab-based medium containing a cocktail of 23 common pesticides revealed selection for mutations conferring streptomycin resistance [129]. In the same study, co-exposure of *E. coli* to pesticides and ampicillin selects for cross-resistance to ciprofloxacin, tetracycline and chloramphenicol, possibly due to mutations in transcriptional regulators responsible for oxidative stress defence or biofilm formation [129].

While it is clear that pesticides have the potential to directly drive AMR evolution, it is also possible that pesticides could shape AMR indirectly, through disrupting soil microbial communities. By reducing community diversity, pesticides can leave communities open to invasion by pesticide- (and antibiotic-) resistant species. These dynamics are frequently reported in the gut microbiota, where antibiotic treatment leaves the gut microbiota vulnerable to invasion by antibiotic-resistant *Clostridium difficile* [130]. Hence, the non-targeted biocidal effect of pesticides, alongside selection for AMR, could create a perfect recipe for a rapid sweep of ARGs through soil communities.

Given the accumulating evidence of pesticide use on the dissemination of AMR, it is important that policy is implemented to prevent further AMR spread and evolution. To address this issue, several solutions can be implemented. First, promoting integrated pest management practices that combine various eco-friendly methods, such as crop rotation, biological control and mechanical pest control, can help minimize pesticide reliance. Second, research into the development of pesticides targeted to a specific pest could significantly lessen the indirect impact on soil microbiota while still effectively controlling pests. Insect viruses, such as baculoviruses, are highly specific in their host range and could be used to target a single insect pest [131]. Plant pathogenic fungi can be controlled by adding soil that contains microbes antagonistic to fungal pathogens. For example, species of *Trichoderma* have been used to control fungal plant diseases caused by *Fusarium* and *Rhizoctonia* [132]. Finally, implementing buffer zones around agricultural fields can also act as a safeguard, preventing pesticide runoff into adjacent ecosystems. By adopting these proactive strategies, we can mitigate the adverse effects of pesticide use on soil microbial communities and reduce the risk of AMR emergence, fostering a more resilient and sustainable agricultural landscape.

## Increased salinity

Climate change brings increased levels of evaporation, low rainfall and global sea level rise [133]. The influx of salts into soils through flooding, alongside a reduction of natural irrigation to remove existing salt, has led to the salinization of one-third of the world’s arable land [134–136]. Bacteria respond to high salinity via (i) increased expression of inorganic ion membrane transporters, porins, and efflux pumps associated with Na^+^, Cl^−^, and K^+^ influx and efflux; and (ii) increased biosynthesis or uptake of compatible solutes – which are compounds that mimic the osmotic properties of salts [137, 138]. Both mechanisms ultimately balance the osmotic gradient across the cell membrane, preventing an influx of salt, which can damage cellular machinery.

High-saline environments have been associated with increased antibiotic production by resident microbes. For example, studies on rhizobia in mangroves show gradients of increasing salinity are associated with increased abundance of antibiotic biosynthesis genes encoding streptomycin (*rffG*, *rffH* and *ISYNA1*); monobactams (*met3*, *asf*, *LysC*); carbapenem (*proA*, *proB*); and penicillin (*pac*) [139]. Conversely, AMR genes similarly increased in abundance under high versus low salt. For example, high salinity levels increased abundance of *cusS* and *copS* (causing reduced membrane permeability), as well as AbcA and BmrA efflux pumps conferring β-lactam resistance [139]. One study confirmed a direct link between increased salinity and the maintenance of AMR genes in soil communities: by performing quantitative PCR on cattle manure microbial communities treated with increasing salt concentrations, Li *et al*. [140] found that high salinity can prevent the loss of AMR genes *tetM*, *sul1*, *ermB* and *intI1*, but not *tetV*, *mexk* or *bacA* [140]. Hence, AMR genes can persist in high-salinity environments, despite the energetic cost of maintaining AMR genes in the absence of antibiotics [140].

Cross-resistance or co-regulation of salt resistance and AMR genes can enhance AMR under high salinity. For example, the EnvZ/OmpR membrane-bound histidine kinases are chemoreceptors which can detect changes in external osmolarity [141]. These receptors regulate the expression of cell permeability porins, OmpC and OmpF, as well as the AcrAB–TolC multi-drug-resistance efflux pump [107, 141–145], enhancing both salt tolerance and AMR [146]. In *Listeria monocytogenes,* salt stress similarly increases the expression of LiaR – a response regulator involved in cell envelope stress and toxic ion responses [147, 148]. Deleting the *liaR* gene increases the sensitivity of *L. monoctytogenes* to nisin [147]. While such studies do not go as far as demonstrating that salt stress drives AMR evolution, they suggest that elevated salt stress could potentially select for *de novo* mutants that have reduced antibiotic susceptibility. An experimental evolution approach could reveal how physiological responses to salt stress feed into selection for AMR over evolutionary time.

MGEs can co-select for ARG in high-salinity environments. Metagenomic analysis of manure samples treated with increasing salt concentrations revealed that high-salinity environments maintain AMR genes *tetM*, *sul1* and *ermB,* because they resided on MGEs containing salt stress resistance mechanisms [140]. Similarly, other salt-resistant mechanisms, such as the biosynthesis of the compatible solute ectoine and the expression of K^+^ transporters [124, 125], have been found on plasmids isolated from halophilic (‘salt-loving’) strains. The loss of such plasmids can alter the sensitivity of halophiles to antibiotics, including ampicillin and vancomycin [144]. Conversely, studies on saline soils have revealed a reduction in ARGs and MGEs under high- compared to low-salinity soils [149]. This was probably caused by a fitness cost of plasmid carriage under salt stress, as well as the loss of important ARG carriers such as *Actinobacteria* in highly saline soils [149]. Hence, the impact of increasing salinity on AMR depends on opportunities for co-resistance (i.e. whether salt-resistance and AMR genes are located on the same MGE) as well as the costs of MGE carriage in a particular environment.

The salinization of arable land is a growing concern, as land use intensifies amid the growing impact of climate change. In order to reduce or maintain soil salinity levels and prevent AMR dissemination, improved irrigation practices are required. This could be achieved by using efficient irrigation methods such as drip irrigation or precision agriculture; farmers can reduce water wastage and prevent the build-up of salts in the soil [150]. However, this approach may not be suitable for arid regions (where the problem is most prevalent) or may be cost-prohibitive for some farmers. Furthermore, promoting soil conservation practices, such as mulching and cover cropping, can help retain soil moisture and reduce salinity levels. However, the effectiveness of these practices depends on local climatic conditions and farming traditions, making adoption challenging in some areas. Salinization is regarded as a serious form of soil degradation and is estimated to be a major challenge for sustaining plant and animal life in the coming decades [133, 151]. Hence, the myriad benefits of employing mitigation measures to protect soils against salinization range from tackling AMR to safeguarding the future production of food.

## Perspectives and future work

Anthropogenic activities have increasingly led to accelerated climate change, environmental pollution and the disruption of natural ecosystems. These events have been identified as potential contributors to the proliferation of AMR [152]. Emerging evidence suggests that anthropogenic actions resulting in increased temperatures, salinity and chemical influx could reduce or negate the cost of AMR in soil microbes. This finding is important because it becomes more difficult to control or reverse AMR if resistance mechanisms do not carry a cost (or are beneficial) in the absence of antibiotics. Worryingly, although antimicrobial stewardship programmes are becoming more successful, global population numbers and food production are becoming more intense. Together, this suggests that without a one-health approach to fighting AMR, intensive agriculture in the face of climate change could negate the positive efforts of antimicrobial stewardship programmes. Below, we highlight three areas of research that should be prioritized:

- A myriad of studies linking bacterial stress responses to AMR stem from gene expression studies of knockout mutant strains lacking stress response gene(s) (e.g. sigma factor σ32 in *E. coli *). The question remains about how relevant short-term stress responses are (which often temporarily enhance tolerance or antibiotic resistance) to long-term AMR selection. Moreover, the knowledge we have gained by studying stress responses in a single species (typically *E. coli *) should now be expanded to gauge the role of stress response systems in natural microbial communities, where the cost and benefits of stress responses are likely to change [153]. Semi-natural experimental systems (e.g. [45]) are useful for testing the responses of a focal species (or community) to a stressor over both short and long timescales in a controlled laboratory setting.
- While our review focuses on how anthropogenic stress can drive AMR, trade-offs could equally influence resistance evolution. However, few studies have investigated trade-offs between mechanisms other than those targeting different clinical antibiotics (i.e. collateral sensitivity [154–156]). Using an experimental evolution approach, Vasse *et al.* [157] demonstrated that antibiotic resistance evolution in *P. aeruginosa* comes at a population-level cost by selectively favouring siderophore 'cheats' that do not bear the cost of siderophore production but reap the metal-chelating benefits of siderophores produced by others [157]. These results indirectly imply that AMR might trade-off with metal chelation (and detoxification). Although there is strong evidence for trade-offs in experimental populations of bacteria [91], whether such trade-offs occur in metal-polluted communities – and influence the spread of AMR genes – remains unclear and is a worthwhile avenue for future research.
- Our review highlights the profound role of natural environments in shaping AMR, and follows several excellent reviews in this area [72, 158]. However, the role anthropogenic stressors (other than antibiotic overuse) could have in driving AMR typically does not reach policy – where the focus is on managing antibiotic use, mainly in the clinic. A recent report by the World Health Organization [159] outlined 40 key research topics for informing policy on AMR – however, agriculture was not mentioned anywhere in this report. There remains a worrying assumption that tackling AMR begins and ends in the clinic, a perception that should now be challenged with translatory research that transcends from science into policy. Opportunities to second or partner research scientists into the public service (such as Science foundation Ireland’s Public Service Fellowship Programme) could allow researchers to bring their expertise to policy-making and innovation at a national and international level.
